# The Epigenetic Modulation of Cancer and Immune Pathways in Hepatitis B Virus-Associated Hepatocellular Carcinoma: The Influence of HBx and miRNA Dysregulation

**DOI:** 10.3389/fimmu.2021.661204

**Published:** 2021-04-29

**Authors:** Kurt Sartorius, Ping An, Cheryl Winkler, Anil Chuturgoon, Xiaodong Li, Julia Makarova, Anna Kramvis

**Affiliations:** ^1^ Hepatitis Virus Diversity Research Unit, School of Internal Medicine, University of the Witwatersrand, Johannesburg, South Africa; ^2^ Department of Public Health Medicine, School of Nursing and Public Health, University of KwaZulu-Natal, Durban, South Africa; ^3^ Department of Surgery, University of KwaZulu-Natal Gastrointestinal Cancer Research Centre, Durban, South Africa; ^4^ Basic Research Laboratory, Frederick National Laboratory for Cancer Research, National Cancer Institute, Frederick, MD, United States; ^5^ Discipline of Medical Biochemistry, School of Laboratory Medicine and Medical Sciences, College of Health Science, University of KwaZulu-Natal, Durban, South Africa; ^6^ Department of Oncology, The Third Affiliated Hospital of Soochow University, Changzhou, China; ^7^ Department of Nutrition and Food Hygiene, School of Public Health, Soochow University, Suzhou, China; ^8^ Faculty of Biology and Biotechnology, National Research University Higher School of Economics, Moscow, Russia; ^9^ Higher School of Economics University, Moscow, Russia

**Keywords:** hepatitis B virus-associated hepatocellular carcinoma, epigenetic modulation, HBx, microRNA, immunology

## Abstract

Hepatitis B virus (HBV)-associated hepatocellular carcinoma (HBV-HCC) pathogenesis is fueled by persistent HBV infection that stealthily maintains a delicate balance between viral replication and evasion of the host immune system. HBV is remarkably adept at using a combination of both its own, as well as host machinery to ensure its own replication and survival. A key tool in its arsenal, is the HBx protein which can manipulate the epigenetic landscape to decrease its own viral load and enhance persistence, as well as manage host genome epigenetic responses to the presence of viral infection. The HBx protein can initiate epigenetic modifications to dysregulate miRNA expression which, in turn, can regulate downstream epigenetic changes in HBV-HCC pathogenesis. We attempt to link the HBx and miRNA induced epigenetic modulations that influence both the HBV and host genome expression in HBV-HCC pathogenesis. In particular, the review investigates the interplay between CHB infection, the silencing role of miRNA, epigenetic change, immune system expression and HBV-HCC pathogenesis. The review demonstrates exactly how HBx-dysregulated miRNA in HBV-HCC pathogenesis influence and are influenced by epigenetic changes to modulate both viral and host genome expression. In particular, the review identifies a specific subset of HBx induced epigenetic miRNA pathways in HBV-HCC pathogenesis demonstrating the complex interplay between HBV infection, epigenetic change, disease and immune response. The wide-ranging influence of epigenetic change and miRNA modulation offers considerable potential as a therapeutic option in HBV-HCC.

## Introduction

In the Globocan 2018 report, 841 080 new liver cancer cases were diagnosed with hepatocellular carcinoma (HCC) (1). Chronic hepatitis B virus (HBV) infection, which has been etiologically implicated in 43% to 80% of total HCC incidence (1–3) remains a primary risk factor and high HBsAg seroprevalence (>5%) levels currently persist in Western Pacific, Africa, East Mediterranean, and East Asia (2). The downstream effect of widespread HBV infection could precipitate 5 million deaths by 2030 from HBV-HCC (3). HBV-HCC pathogenesis is fueled by persistent HBV infection that stealthily ensures a careful balance between replication and the need to remain under the “radar” of the host immune system. HBV is remarkably adept at using a combination of both its own, as well as host machinery to manage viral load and enhance persistence (4, 5). A key tool in this regard, is the manipulation of the epigenetic landscape to modulate the silencing role microRNA (miRNA). The role of HBV dysregulated miRNA at each stage of the HBV-HCC continuum has been well documented from the onset of HBV infection to fibrosis/cirrhosis and the onset of HBV-HCC (6). The regulatory role of HBV dysregulated miRNA has also been extended to specifically examine the interplay between HCC and immune pathways (7).

This review examines the epigenetic role of HBx dysregulated miRNA in HBV-HCC pathogenesis. HBx is a 17 kDa transactivating protein expressed from the *X* open reading frame of HBV, with little sequence homology to any known genes, hence the name “X”. HBx can modulate several hepatocyte signaling cascades and factors associated with mechanisms that induce cellular transformation. Unlike mammalian hepadnaviruses (HBVs), avian HBVs do not express HBx and do not develop HCC leading to the postulation that the HBx protein has oncogenic potential (8, 9). HBx, can initiate a wide range of epigenetic changes implicated in hepatocarcinogenesis including DNA methylation, histone modifications, chromatin remodeling and microRNA (miRNA) dysregulation (10). One of these epigenetic factors, namely, miRNA are themselves influenced by epigenetic modulation often forming feedback loops that modulate epigenetic change (11, 12).

We attempt to link the epigenetic role of HBx dysregulated miRNA in HBV-HCC pathogenesis in both the HCC and immune pathways. The review commences by describing how the HBx viral protein can modulate both the host, as well as HBV genome expression by influencing the epigenetic landscape. Next a comprehensive list of HBx dysregulated miRNA are presented in **Table 1**, which are influenced by upstream epigenetic changes, and/or their downstream epigenetic targets. This table also identifies host gene targets in both the HBV-HCC cancer and immune pathways. Four comprehensive examples of epigenetically modified miRNA are then outlined in the next section to demonstrate the complex interplay between viral infection, HBV-HCC pathogenesis and immune system response. The review then examines the potential therapeutic role of miRNA that has yet to be deployed outside of the laboratory setting.

**Table 1:** HBx-dysregulated epi-miRNA and their targets in HBV-HCC.

HBx-epi-regulator	miRNA	Epi target	HBV-HCC gene target	Immune gene target	Epi-Ref	HCC Reference
HMT/EZH2 LIN28/tet1/EED/SUZ12	Let-7c	SUZ12/EED/EZH1/2	*CNKD1/PRICKLE/SFRP5/B-CAT/STAT3/RAS/HMGA2/MYC/ IL-6/IL-10/TLR-4/COL1A2/NGF/BCL-XL/BCL-2/MCL-1z*	*MYC/STAT3/IFN-b/RAS/TLR4 BCL-XL/SMAD2/SMAD4/NFZ APC2/WNT1/HMGA2/PLZF/IFN/IL-4/IL-17/LIN28B/IGF2BP1*	(13–15)	(16–19)
DNMT	miR-1	HDAC4	*EDN1/PI3K/AKT/METFOXP1*	*E2F5/HSP60/HSP70/KCNJ2/GJA1*	(20–22)	(20, 21, 23)
HMT/EZH2/EED/C-MYC/SUZ12	miR-101	DNMT3A/EZH2/EED/SUZ12/	*GSTP1/FOS/MCL-1/RASSF1A/PRDM2/CNKD1/PRICKLE/SFRP5/B-CAT/AP1/DUSP1/MCL-1/ROCK2/ATG4D/MTOR/SOX9/COX2/RAB5A/STMN1/DNMT3A/FOS/RAP1B/VEGF*	*ICOS (naïve T-cells)/MCL-1*	(13, 14, 24)	(25) (14, 24, 26–28)
DNMT/PPARα	miR-122		*CTNNB1/CCNG1 modulated p53/GLD2/NDRG3/GALNT10/CCNG1/PTTG1/PBF/ADAM10/CCNG1/Igf1R/ADAM 17/BCL-W/NDRG3*	*SOCS3/IFN/IP-10/BCL-W*	(29, 30)	(31–34)
DNMT1	miR-124	EZH2/BMI1	*STAT3/PIK3CA/ROCK2/STAT3/Cyclin D/*CDK6, VIM, SMYD3, E2F6, IQGAP1	*STAT3/TRAF6/CYCLIND3/BM11*	(14, 35, 36)	(37)
	miR-125a	SIRT7	*MMP11/VEGF-A/ERBB2/HBsAg*	*NF-α/BCL-2/KLF13/BMF*	(38, 39)	(40–42)
EZH1/2/HMT/SUZI2/EED/p53	miR-125b	SIRT7/SUZI2 SUV39H1/EED/EZH1/EZH2/	*SMAD2/4/Sirtuin7/SUV39H1/LIN28 B/PIGF/BCL-2/MCL-1/CNKD1/PRICKLE/SRFRP5/B-CAT/*PIGF/MMP2/MMP9	*PRDM1/IRF4/TNF_/BCL-2/MCL-1/LIN28/IRF4/KLF13/BMF/BCL-2/SMAD2/SMAD4 APC2/WNT1//KLF13/TRP5 3INPI/LIN28A/IRF4/BLIMP1 IRF4/BMF*	(13, 36, 43, 44)	(45, 46)
DNMT3	miR-132	p300	*AKT/GSK3/WNT-BCAT*	*p300/IRAK4/FOXO3/SOX4/*	(47, 48)	(48)
HMT/EED/SUZ12/EZH2	miR-139-5p	EED/SUZ12/EZH1/2	*ZEB1/2/CNKD1/PRICKLE/SFRP5/B-CAT*	*IL-4/IFN-γ*	(13, 14)	(45, 49, 50)
	miR-140	DNMT1	*NF-kB/TGFβRI/FIF9/Pin1*		(51)	
	miR-145	HDAC2	*MAP3K/CUL5/ADAM17*	*IFN-b/TIRAP/TRAF6*	(52–54)	(55–57)
DNMT1/p53C-MYC	miR-148a	DNMT1	*HPIP/AKT/ERK/FOXO4/ATF5/ERBB3/BCL-2/IRS-1/MTOR/MET/ACVR1/SNAIL/IGF-IR/MIG6/CAND1/CDC25B*	*CaMKIIα/KIT/MET/SIPI/BACH/PTEN/BIM/GADD45*	(58, 59)	(45, 60–62)
	miR-152	DNMT1/DNMT3A	*GSTP/CDH1/KIT*	*CaMKII/KIT*	(22, 47, 63)	(42, 63–65)
HDAC-I/EZH2	miR-155	PRC2/Phf19/p300/CBP	*PTEN/SOX6/ZHX2/SOCS1*	*AID/Blimp-1/PRDM1/IFN/SHIP1/SOCSI/BMAL1/PU.1/BACH1/CSFIR/CEBP/ETS1/Th2 induction3/SOCS1/C/EBP/AID/FOXP3*	(43, 66, 67)	(66, 68, 69)
HDAC-I SAHA/ C-MYC	miR-17-92 family	DNMT	*E2F1, Cyclin G1/PTEN/p21/p27/p57/cccDNA*	*TNFSF9/CCL-5/IKBKE/c-MAF/AMLI/TP53INPI c-MAF/IFN/CD69/PTEN/TGFBR11/p27/p21/E2F/PHLPP2/BIM/CREB1*	(70, 71)	(72–74)
HMT	miR-199a/b		*CHC*	*CD19+*	(75)	(76)
DNMT/p53	miR-200a	HDAC4	*ZEB1/2/HNF-3β Rho/ROCK/ASB4*		(77, 78)	(45, 79)
EED/SUZ12/EZH1/2	miR-200b	EED/SUZ12/EZH1/2	*CNKD1/PRICKLE/SFRP5/B-CAT*		(13, 78)	(45, 79)
EZH2/BMI1	miR-203		*RAP1A*	*SMAD1/BCL11B/RARB/PRKCA/PRKCB1/FMRP*	(80)	(81)
DNMT	miR-205		*ACSL4/E2F1/ZEB1/2*		(82)	(45, 82, 83)
DNMT	miR-221	HDAC6	*ERα/DDIT4/BMF/p27 p57/PTEN/p21/SOCS3*	*PTEN/SOCS3/p57/KIT/p27*	(84, 85)	(86–88)
DNMT	Mir-222		*P27kip 1/PTEN/PPP2R2A/p57/p21*	*p27 kip 1/PTEN/KIT*	(85)	(55, 88–90)
HAT/HDAC1/3/EP300/p50/p65	miR-224		*PAK4/MMP9 inhibitor-5/SMAD4*	*AP15/SMAD4*	(91, 92)	(93–95)
	miR-26a	EZH2	*IL-6/IFN_/ER_/Cyclin D2/Cyclin E2/c-JUN/CDK4/6*	*IFN-b CDK4/6/MALT1*	(96–98)	(99–101)
	miR-29c	DNMT3B/SIRT1	*BCL-2/MCL-1/TNFA1P3*	*TCL-1/MCL-1/IFN-*	(102–104)	(105, 106)
H3K4ac	miR-29a/b	DNMT1/DNMT3A/B/SETDB1/H3K9/SIRT1	*PTEN/PI3K/AKT/MMP-2*	*IFNARI/IFN/T-Bet/EOMES/PTEN/MCL-1/IFN-/SLFN4/CDC42/HBP1/TCL1*	(22, 102, 103, 107, 108)	(55, 109)
HBx/P53/DNMT1/3A/3B	miR-34a	SIRTI	*CCL22/MAP4K4/SIRT1/CCND1/CDK4/6/MET/C-JUN/CDK2*	*IFN-b/FOXP1/CDK2/4/6/CCL22/FOXPN*	(110)	(111–115)
HDAC	miR-373	HDAC/SIRT1	*SNAIL-1/CDH1*	*MTOR/SIRT1/RELA*	(116)	(116)
	miR-548a	HDAC4	*HBXIP, IFN-λ1*	*IFN-λ1*	(117)	(118, 119)

Column 1 indicates upstream epigenetic proteins that can influence miRNA expression in column 2 (blue miRNA- downregulated and red miRNA-upregulated). Column 3 is downstream epigenetic targets of dysregulated miRNA. Columns 4 and 5 are specific HBV-HCC and immune gene targets. Column 6 refers to epigenetic references only (column 1 and 3) and column 7 to HBV-HCC and immune gene targets.

## HBV-HCC Pathogenesis: Role of miRNA

Persistent HBV infection remains a global risk factor that can promote the development of fibrosis/cirrhosis and ultimately the onset of HBV-HCC (120). A significant component of HBV-HCC pathogenesis involves the integration of HBV DNA into the host genome that results in the oncogenic disruption of cellular genes (121). HBV DNA integration can cause host cell deletions, *cis*/*trans*-activation, translocations, the increased production of fusion transcripts, aberrant epigenetic changes and generalized genomic instability (122). These changes take place in the form of chronic inflammation and tissue damage that result in the continuous destruction of well differentiated hepatocytes and organized extracellular matrix (ECM). Over time the depletion of hepatocytes and well organized ECM results in their replacement with undifferentiated liver stem cells and poorly organized fibrotic tissue (120) that display changing patterns of apoptosis, regeneration, senescence and survival (123). HBV-HCC pathogenesis involves the deregulation of many cellular signaling pathways including the Wingless-related integration site/Beta-Catenin (WNT/β-CAT), in the Retinoblastoma-Tumor Protein 53 (RB1-TP53) suppressor networks, the Phosphoinositide 3-kinase/mitogen-activated protein kinase (PI3K/MAPK), the Janus kinase/signal transducer (JAK/STAT) pathways and the insulin receptor substrate-1/insulin growth factor (IRS1/IGF) pathways (124–126).

MicroRNA (miRNA) are a subsidiary subset of epigenetic factors that act as post-transcriptional gene silencers in the HBV-HCC pathways. miRNA collectively attempt to repress target mRNA expression in order to ensure homeostasis and their fluctuating role is explained by the inherently stochastic nature of gene transcription and environmental fluctuations (127). In the HBV-HCC continuum, from asymptomatic HBV infection leading to HCC, an increasing number of miRNA are dysregulated due to the need to respond to viral infection, epigenetic changes (35), inflammation (128), fibrosis (129), cirrhosis (123) and finally, the onset of HCC. One of the most documented HBV tools to modulate both its own, as well as host genome expression is the HBx protein which has been shown to dysregulate multiple miRNA species in key HCC cancer and immune pathways (7).

## HBx Induced Epigenetic Changes in HBV-HCC Pathogenesis

**Figure 1:**
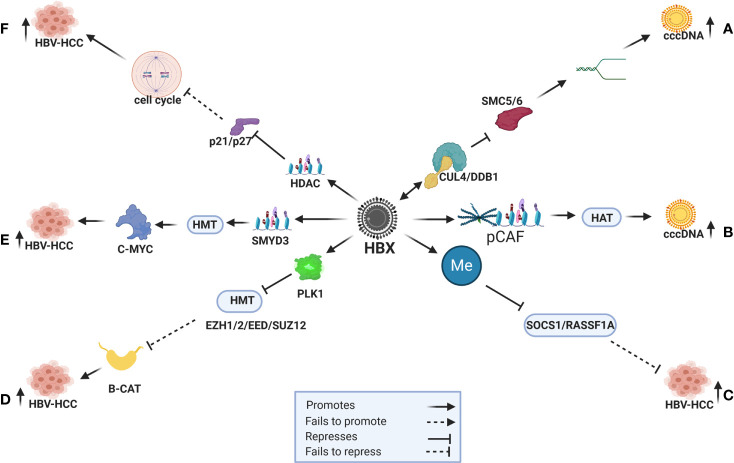
HBx induced epigenetic expression in HBV-HCC. **(A)** HBx recruits the CUL4/DDB1 ubiquitination complex to repress SMC5/6 and promote DNA relaxation and cccDNA transcription. **(B)** HBx recruits CBP/p300 (pCAF) to transactivate HAT (H3K9ac) to promote cccDNA expression. **(C)** HBx promotes DNMT to repress SOCS1/RASSF1A tumor suppressors that fail to modulate HCC pathogenesis. **(D)** HBx promotes PLK1 to block HMTs (EZH1/2/EED/SUZ12) that then fail to modulate B-CATENIN induced hepatocarcinogenesis. **(E)** HBx initiates HMTs (SMYD3) to promote C-MYC induced HCC. **(F)** HBx promotes HDAC to silence cell cycle controls like p21/p26 and promote HCC.

HBV DNA and its proteins influence own and host genome expression by employing a range of epigenetic modifications in HBV-HCC pathogenesis, as well as modulating signal transduction, transactivation and transcription to regulate immune response, cell cycle, apoptosis and DNA repair (130). In particular, the HBx protein influences DNA methylation (131), histone modifications (132), chromatin remodeling (133) and miRNA dysregulation (6) which is the central focus of this review (**Figure 1**).

### Regulation of cccDNA Activity

The HBV covalently closed circular DNA (cccDNA), a stable mini-chromosome that is classified as nuclear episomal DNA, serves as the template for viral RNA transcription. In order to enhance the efficiency of HBV replication, this virus co-opts host transcription machinery like HNF1/2/3, C/EBP, CREB and CRTC1 to trigger cccDNA transcription (4). The HBx protein appears to be co-opted to influence its own transcription by triggering histone modifications including histone acetylation and deacetylation (HAT/HDAC), histone methylation and demethylation (HMT/HDMT), DNA methylation (DNMT) and ubiquitination (5). An example in this respect, is when cccDNA transcription is increased the HBx protein co-opts DDB1 and Cul4 to create a ubiquitination complex to repress SMC5/6 which maintains the structure of chromosomes. The repression of SMC5/6, therefore, promotes DNA relaxation to increase the transcription of cccDNA (131, 134) (**Figure 1A**). The HBx protein can also increase cccDNA transcription by recruiting CBP/p300 (pCAF) to transactivate HATs (H3K9ac) that leads to hyperacetylation (131, 135) (**Figure 1B**).

### DNA Methylation

DNA methylation, the addition of a methyl group by the enzymes DNMT1/2/3A/3B, can silence target gene promoters or enhance target gene expression by silencing transcription regulators in both the HBV and host genome (4, 5). In percentage terms, common host genes targeted for DNA methylation in HBV-HCC include WT1 (54%), SOCS1 (43-65%), SEMA3B (83%), RASSF1A (59-75%), p300 (65%), p27 (48%), p21 (63%), p16 (16-83%), GSTP1 (41-76%), E-cadherin (33-67%), E2F1 (70%), CPS1 (80%) and APC (53-81%) (10). In many cases these epigenetic changes silence tumor suppressor or activate oncogenic proteins and the HBx protein influences DNMT expression to subdue the host immune response, as well as manage its own expression to promote a balance between survival and replication (4). The HBx protein, for example, often promotes DNMT1/3A/3B expression to silence tumor suppressor genes like SOCS1/RASSF1A in HBV-HCC pathogenesis (4, 10) (**Figure 1C**). It can also use this machinery to suppress cccDNA expression in order to evade the host immune system (136).

### Histone Modifications

Histone modifications are post translation modifications to histone proteins that include HAT/HDAC, HMT/HDMT, phosphorylation and ubiquitylation. These post translational modifications primarily occur at the N-terminal of the histone tails and have a fundamental impact on chromatin remodeling which essentially alters the structure of host and HBV DNA in HBV-HCC pathogenesis. For example, the HBx protein can promote HAT by recruiting trans-activator proteins like CBP/p300 complex to induce H3K9ac (135) Conversely, the HBx protein can induce HDAC to promote cell proliferation in HBV-HCC by repressing tumor suppressors like p21/p27 that are important regulators of cell cycle control (132) (see **Figure 1F**) and promote epithelial-mesenchymal transition (EMT) by repressing CDH1 (137). The HBx protein can also use HDAC to modulate the viral genome expression by recruiting HDAC1/2 to cccDNA to suppress its expression (4, 5). In addition, HBx also induces HMT by upregulation of the SMYD3 gene, which encodes a histone H3-K4-specific methyltransferase to trigger oncogene expression (138). The HBx protein can also induce histone methylation transferases and it has been demonstrated that HBx induced upregulation of SMYD3, that encodes for histone H3-K4-specific methyltransferase (HMT), is linked to the upregulation of the oncogene *C-MYC* in HCC (138, 139) (**Figure 1E**). Conversely, the HBx protein can reverse the repressive effect of histone methylation (H3K9me3) on cccDNA by initiating a histone demethylation (HDMT) agent (140).

### Polycomb Proteins

The Polycomb repressive complex (PcG) proteins, namely, Polycomb repressive complex 1 (PRC1) and 2 (PRC2) form part of the histone modification machinery that epigenetically regulate chromatin remodeling. PRC2 participates in histone methylation (H3K27me3) and, following histone H2AK119 mono-ubiquitination by PRC1, collaboratively represses target gene transcription (141). In the HBV-HCC continuum, the HBx protein can co-opt the proteins of these two complexes to influence epigenetic changes. For example, HBx upregulates the proto-oncogene PLK1, an enzyme that can block the repressive effect of the PRC2 complex (SUZ12/EED/EZH1/EZH2) to down-regulate WNT antagonists (CNDK1/PRICKLE/SFRP5). The repression of these WNT antagonists leads to increased β-catenin transcription and hepatocarcinogenesis (13, 142) (**Figure 1D**).

## HBx Dysregulated lmiRNA in HBV-HCC and Epigenetic Change

In the HBV-HCC continuum, the HBx protein can influence epigenetic changes like DNA methylation, histone modifications and other non-coding RNA that dysregulate a specific subset of miRNA (called epi-miRNA) that forms the central focus of this review (**Table 1**). In addition, the HBx protein can dysregulate host genes that modulate miRNA biosynthesis, transcription and translation (47). Simultaneously, miRNA expression modulates downstream epigenetic modulation by targeting epigenetic modifiers suggesting epigenetic feedback loops that directly influence both miRNA and their downstream epigenetic targets (4, 10, 45). In the HBV-HCC continuum, HBx-dysregulated miRNA, therefore, are epigenetic regulators that are themselves epigenetically modulated. In **Table 1** we show the HBV-HCC specific gene targets of miRNA identified in the literature, as well as their specific immune targets in HCC. It should be borne in mind that these are dynamic pathways and that the proposed regulatory effect of miRNA is also dynamic.

The HBx protein can upregulate or downregulate specific miRNA expression using specific epigenetic regulation (**Table 1**). These mechanisms, for instance, include DNA methylation of miR-1/-122/-124/-132/-/148/-200/-205 genes to downregulate miRNA expression (24, 48), histone acetylation or HDAC inhibitors to upregulate miR-224/-29/-155/-17-92 and histone methylation to downregulate Let-7c/miR-101/-125b/-139-5p (45). The HBx protein can also target upstream transcription factors essential for miRNA expression like p53 suppression of miR-23a/-34/-125b/-148a/-192/-200 (143) and C-MYC upregulation of miR-15a/-16/-26a/-101/-148a/-363 (144). This protein also targets p50/65 upregulation of miR-143/-224 (93)and NF-_K_B upregulation of miR-143/-146a, as well as dysregulating miRNA expression by repressing miRNA biosynthesis machinery like DROSHA (145, 146).

## HBx-Dysregulated miRNA in HBV-HCC Pathways

**Figure 2:**
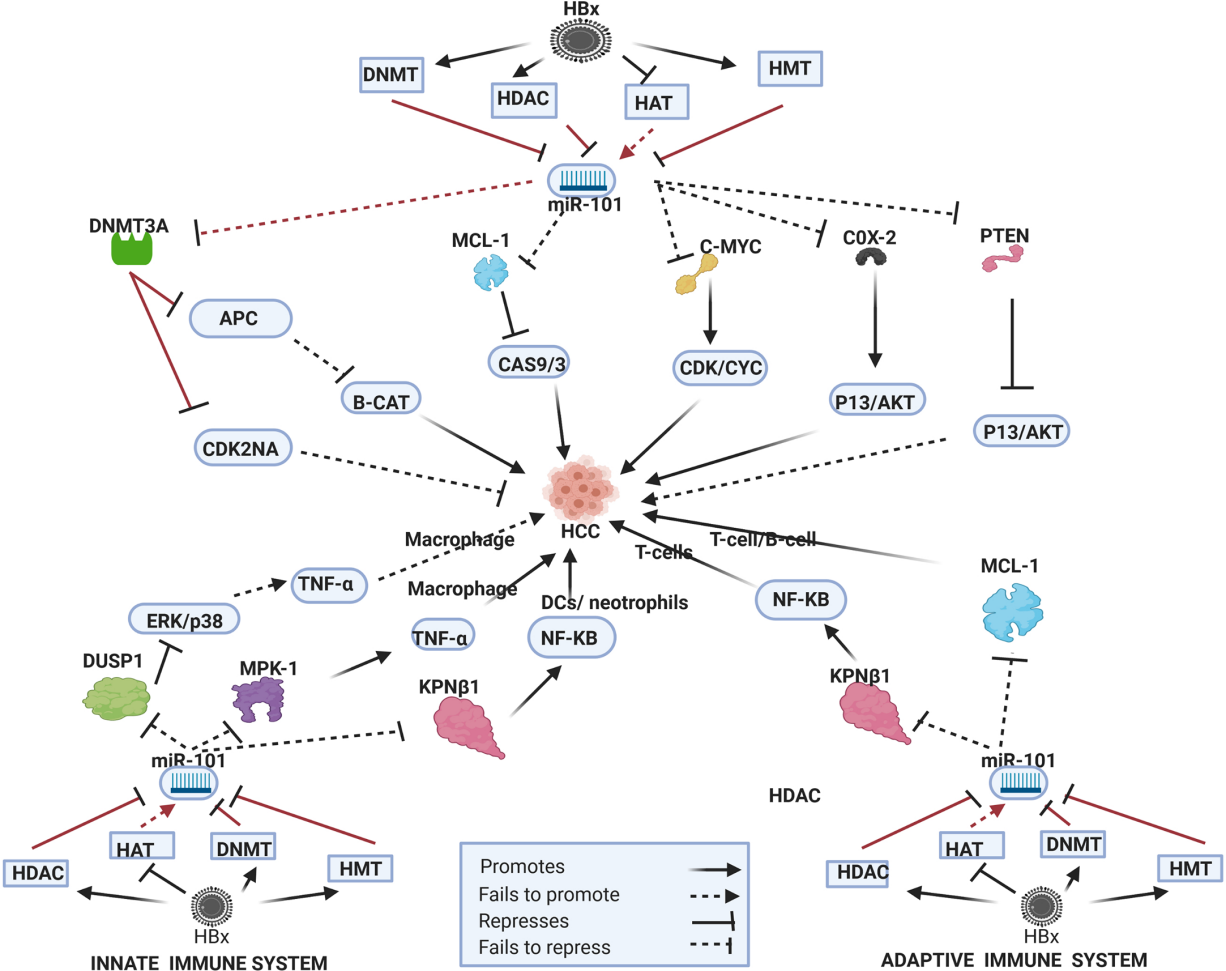
Epi-miR-101 in HBV-HCC and immune pathways. HDAC-Histone deacetylation, HAT-histone acetylation, HMT-Histone methylation, DNMT-DNA methylation (pathways in dark red involve identified direct upstream or downstream epigenetic proteins/enzymes). HBx protein can downregulate or initiate HDAC/HAT/DNMT/HMT to repress miR-101 modulation in various HBV-HCC pathways including WNT-B-CATENIN, TP53, and P13K/AKT to influence HCC pathogenesis. HBx epigenetically downregulated miR-101 also regulates macrophage and DC expression in the innate immune system, as well as T-cell and B-cell expression in the adaptive immune system.

**Figure 3:**
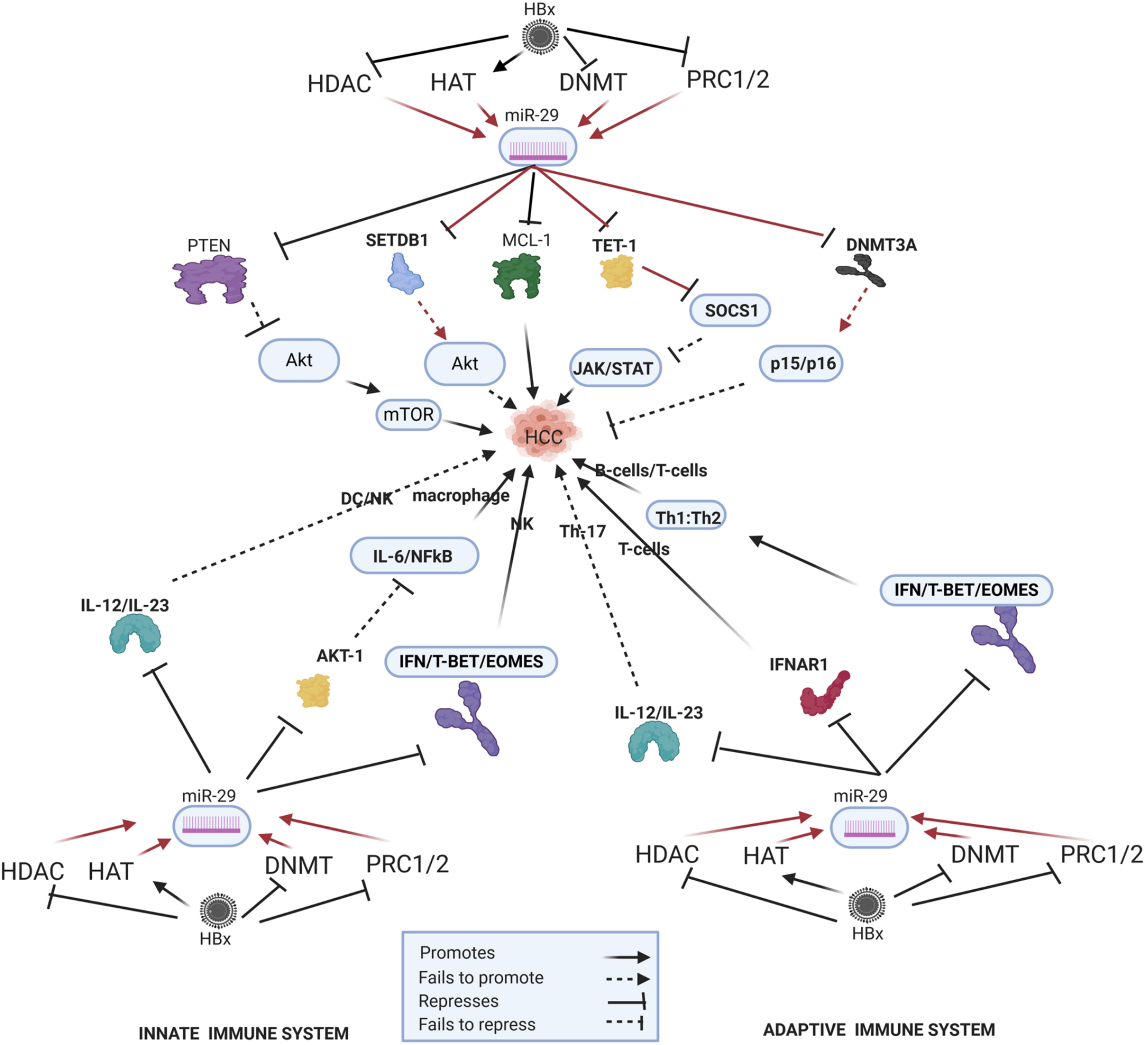
Epi-miR-29 in HBV-HCC and immune pathways. HDAC-Histone deacetylation, HAT-histone acetylation, DNMT-DNA methylation, PRC1/2- Polycomb Repressive Complex, SETDB1-SET Domain Bifurcated Histone Lysine Methyltransferase, TET1-Ten-eleven translocation methylcytosine dioxygenase 1 (pathways in dark red involve identified direct upstream or downstream epigenetic proteins/enzymes). HBx protein can downregulate or initiate HDAC/HAT/DNMT/HMT to upregulate miR-29 modulation in various HBV-HCC pathways including AKT/MTOR, TP53, and JAK/STAT to influence HCC pathogenesis. HBx epigenetically upregulated miR-29 also regulates macrophage and DC/NK expression in the innate immune system, as well as T-cell and B-cell expression in the adaptive immune system.

**Figure 4:**
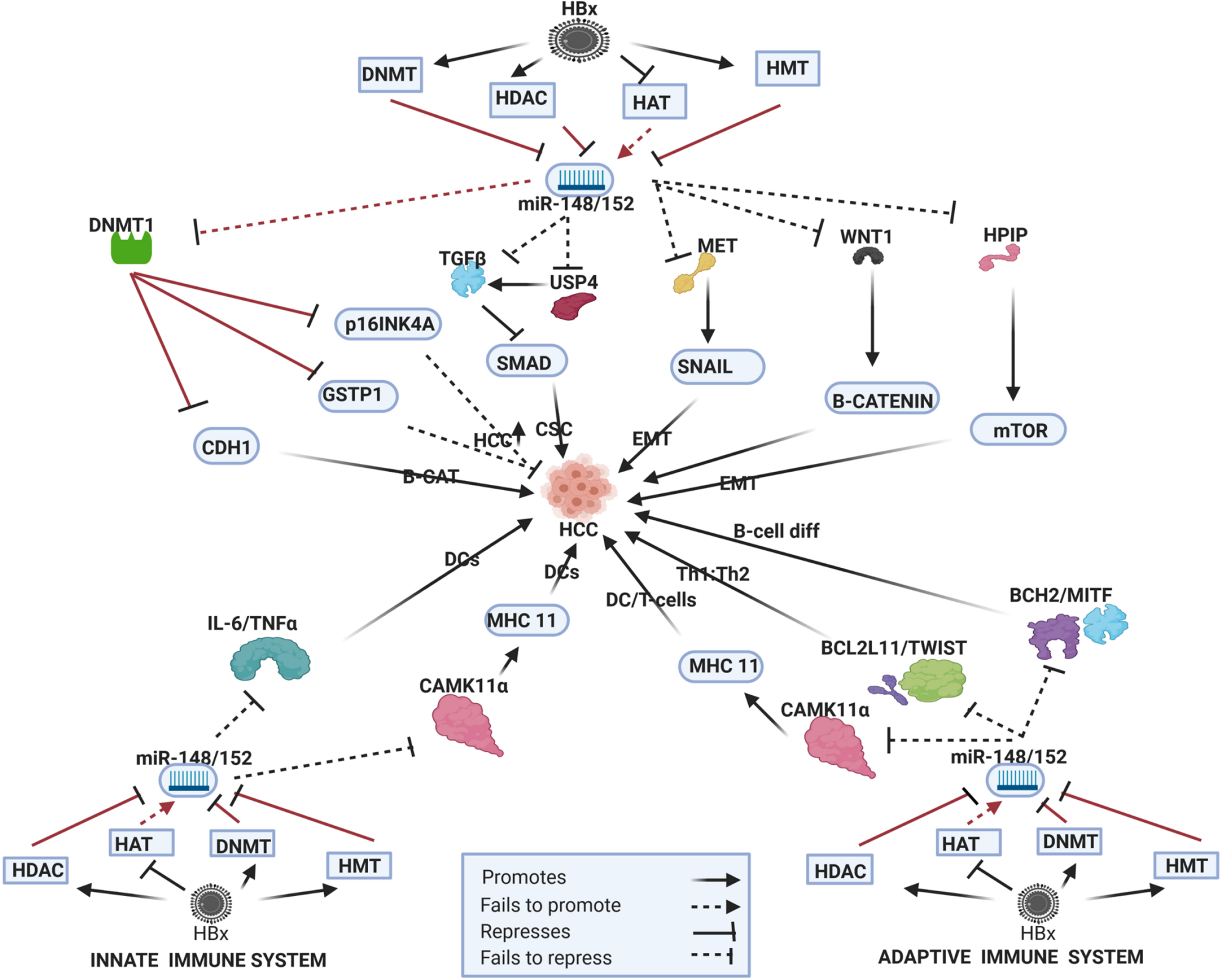
Epi-miR-148/152 in HBV-HCC and immune pathways. HDAC-Histone deacetylation, HAT-histone acetylation, DNMT-DNA methylation, HMT-Histone methylation (pathways in dark red involve identified direct upstream or downstream epigenetic proteins/enzymes). HBx protein can downregulate or initiate HDAC/HAT/DNMT/HMT to repress miR-148/152 modulation in various HBV-HCC pathways including WNT-B-CATENIN, TP53, and AKT/mTOR to influence HCC pathogenesis. HBx epigenetically downregulated miR-148/152 also regulates DC expression in the innate immune system, as well as T-cell and B-cell expression in the adaptive immune system.

**Figure 5:**
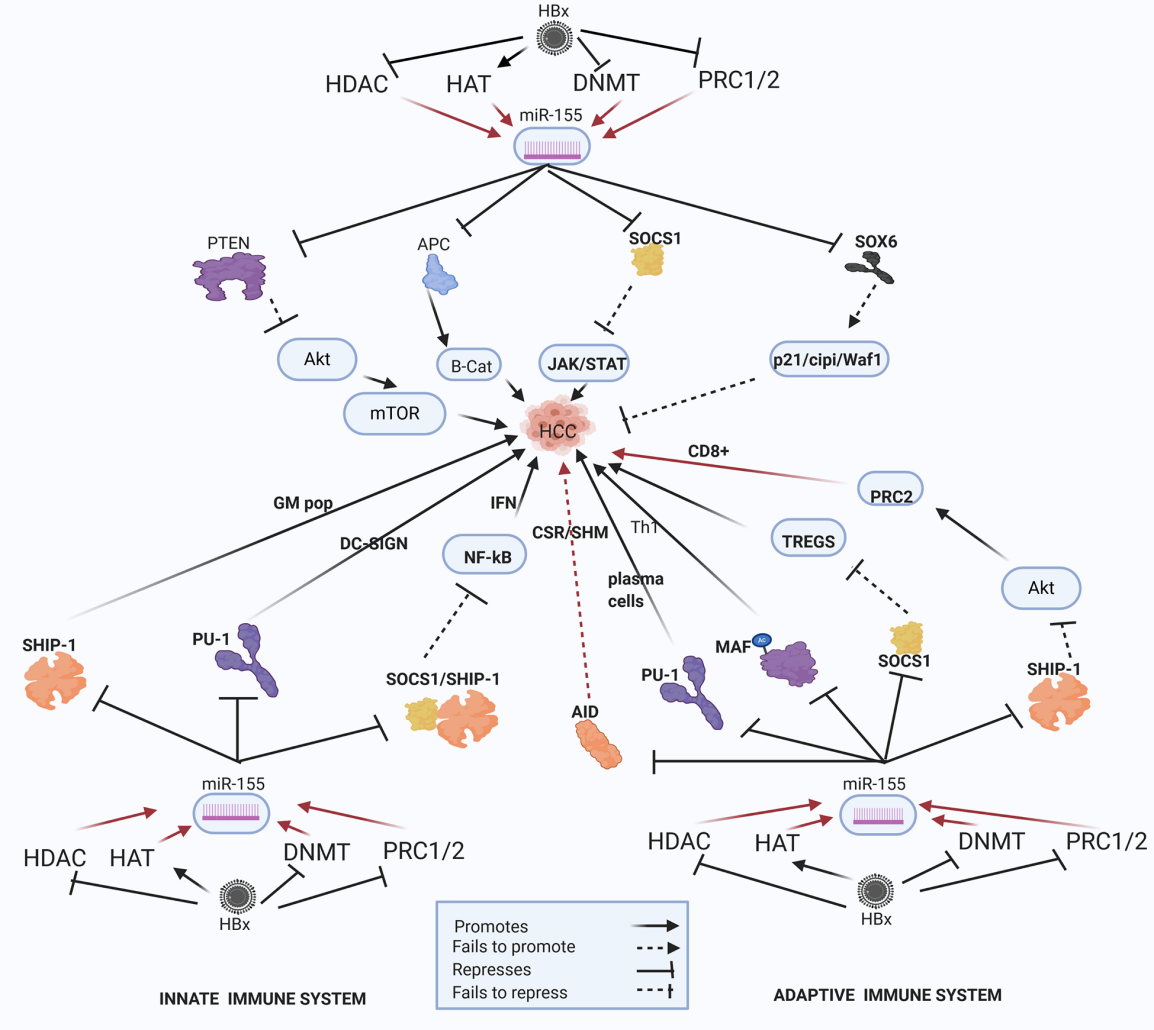
Epi-miR-155 and HBV-HCC and immune pathways. HDAC-Histone deacetylation, HAT-histone acetylation, DNMT-DNA methylation, PRC1/2- Polycomb Repressive Complex 1 and 2 (pathways in dark red involve identified direct upstream or downstream epigenetic proteins/enzymes). HMT-Histone methylation. HBx protein can downregulate or initiate HDAC/HAT/DNMT/HMT to upregulate miR-155 modulation in various HBV-HCC pathways including AKT/MTOR, TP53, and JAK/STAT to influence HCC pathogenesis. HBx epigenetically upregulated miR-155 also regulates germinal matrix (GM) population and NF-kB expression in the innate immune system, as well as T-cell and B-cell expression in the adaptive immune system.

In the HBV-HCC continuum, upregulated miRNA often reduce tumor suppressor expression in the four key HCC cancer pathways, namely, the P13K/MAPK, WNT/β-Catenin, TP53 and JAK/STAT pathways (125). In this section we seek to demonstrate the complexity of the interlocking roles of viral infection, selected epigenetic changes of miRNA in HBV-HCC pathogenesis and the resulting modulation of the host immune system. We illustrate some of the proven epigenetic pathways in HBV-HCC pathogenesis by using four well researched miRNA (miR-29a/b, miR-155, miR-148/152 and miR-101). Two of these miRNA are downregulated (blue) and two are upregulated (red) (see **Figures 2**–**5**). These four miRNA are all HBx epigenetically dysregulated in various HBV-HCC pathways, as well as exercise diverse roles in both the innate and adaptive immune pathways. In many cases, the same miRNA plays a significant regulatory role in many other cancers like those of the breast, lung and colon (147). It is important to highlight that the illustrated hypothetical pathways in **Figures 2**–**5** occur in a dynamic context and that the degree of influence of any single path is non constant. It is also important to keep in mind that the HBx protein can modulate miRNA *via* non epigenetic pathways (e.g. C-MYC/p53) and that in HBV-HCC pathogenesis multiple other factors (e.g. somatic mutations) also influence miRNA expression (45).

### HBx-Dysregulated Epi-miR-101

#### HBV-HCC Pathogenesis

In HBV-HCC and many other cancers, the tumor suppressor miR-101 is regarded as a key miRNA in epigenetic systems (147) (see **Figure 2**). This HBx-dysregulated miRNA involves both upstream and downstream epigenetic systems and has been widely reported as downregulated in HBV-HCC (147). The HBx protein can recruit the Polycomb protein EZH2 (148) to downregulate miR-101 but a feedback loop exists because this miRNA also targets EZH2 (149). In HBV-HCC pathogenesis a demonstrated EZH2 downregulated miR-101 pathway promotes HCC progression as a result of failing to modulate COX-2 activated AKT signaling (14, 150). This downregulated miRNA also fails to block the oncogenic MYCN, a member of the MYC family of proteins that are widely cited in many cancers including HCC. The MYC family can directly promote proliferation by promoting CDK/CYC expression (14, 149, 150) as well as promote angiogenesis *via* promoting VEGFA expression (151). In HBV-HCC pathogenesis, a direct epigenetic target of this miRNA is DNMT3A which targets a range of tumor suppressors. It has been demonstrated that HBx-downregulated miR-101 fails to modulate DNMT3A expression in HBV-HCC and this can contribute to the silencing of tumor suppressor genes like SF1/PRDM2/GSTP1/RUNX3/APC/CDKN2A/STMN1 (24, 149). The silencing of cell cycle inhibitors like CDKN2A in the TP53 pathway, for example promotes cell proliferation in HCC while silencing of the APC tumor suppressor promotes β-catenin expression and the development of EMT (152, 153) HBx (EZH2) downregulated miR-101 can also fail to modulate MCL-1, a key anti-apoptotic member of the BCL-2 family, thus promoting survival in HCC cells as a result of suppressing caspase driven apoptosis (152, 154). This downregulated miRNA also fails to repress the proto-oncogene c-FOS which regulates transcription activity and results in increased invasiveness in HCC pathogenesis (26). EZH2 downregulated miR-101 also fails to modulate *CCDC88A* that codes for the oncogenic protein GIRDIN which regulates many signal transduction pathways such as AKT/PKB, GAI/S, EGFR and is linked to increased migration and invasiveness in HCC (155). Conversely, downregulated miR-101 also targets the tumor suppressor PTEN in the P13/MAPK pathway that contributes to the activation of this pathway and HBV-HCC pathogenesis (156).

#### Innate Immune System

Downregulated miR-101 can downregulate the activation of LPS-stimulated macrophages by failing to modulate MKP-1 which de-activates p38 and JUN induction of pro-inflammatory cytokines like TNF-α (157, 158). In another pathway in the innate immune system, LPS-stimulated macrophages are reduced by HBx repressed miR-101 as a result of its failure to modulate DUSP1 which, in turn downregulates ERK1/2/p38/JNK promotion of pro-inflammatory cytokines like TGF-β (159). Reduced DUSP1 is often noted in HCC, however, and it has been demonstrated that it plays a protective role in HCC by lowering the ERK cascade and thus repressing cell proliferation (160). TGF- β plays a major role in the regulation of inflammatory processes that influence immune cell development (159). Alternatively, miR-101 can play a role macrophage expression *via* activating the NF-_K_B signally as a result of the upregulation of the KPNB1 transport protein (161–163) which is commonly reported in many cancers including HCC (164). In the innate immune system the activation of dendritic cells (DCs) and neutrophils is also directly influenced by NF-_K_B signaling suggesting that miR-101 can potentially play a wider role in the activation of leucocytes (165).

#### Adaptive Immune System

The differential expression of miR-101 has been implicated in modulating T-cell activation. A direct target of miR-101 is the Inducible T-cell co-stimulator (ICOS) mRNA that acts in concert with interaction between T cell receptor(TCR) and MHC class 1 and 2 peptides (166, 167). T-cell function is highly dependent on miR-101 modulation of ICOS mRNA and a reduction in miR-101 mediated regulation can increase ICOS expression on naïve T-cells increases, causing an effector T-cell-like phenotype and that results in autoimmunity (166). HBx-induced stimulation of EZH2 suppresses miR-101 in HBV-HCC (14, 24) and this effect could lead to ICOS upregulation that influences T-cell activation (166). HBx-downregulated miR-101 in HBV-HCC results in a failure to modulate the anti-apoptotic protein MCL-1 thus promoting cell survival (152). Acting alongside this relationship, MCL-1 expression influences an increase in CD8+ T-cell activity (168). A further hypothetical pathway, supported by various studies indicates that miR-101 can initiate T-cell expression, T-cell differentiation and memory T-cells *via* activating NF-_K_B signaling by promoting the expression of the KPNB1 transport protein. KPNB1 is often reported as upregulated in many cancers including HCC (161, 162, 169).

#### B-Cells

HBx-downregulated miR-101 can also influence B-cell development *via* the regulation of MCL-1 which is regarded as a crucial input to B-cell synthesis (170).

### HBx-Dysregulated miRNA-29 Family

#### HBV-HCC Pathogenesis

HBx upregulated miR-29a/b plays a key epigenetic role in modulating aberrant DNA methylation which is often a key feature of HBV-HCC pathogenesis (171) (**Figure 3**). HBx-upregulated miR-29 can repress DNMT3A/3B in HBV-HCC (102) acting as a feedback mechanism to modulate DNA methylation. This can influence a range of downstream effects because DNMT3A/3B often targets cell cycle controls in HBV-HCC including CDKN2A, p16(^INK4A^) and p15(^INK4B^) (171). By repressing DNMT3A/3B, HBx-upregulated expression of miR-29 acts in support of host cell cycle controls. DNMT3A/3B also silences many tumor suppressors in HBV-HCC pathways like RASSF1/PRDM2/GSTP1/RUNX3/APC therefore, miR-29 induced DNMT3A/3B repression contributes to a secondary support system for tumor suppressor expression to slow HCC progression (172). HBx-upregulated miR-29 family members could increase cell survival by targeting BCL-2 proteins (MCL-1) that retard apoptosis by contributing to the down regulation Caspase 9/3 driven apoptosis in the TP53 cancer pathway (105, 173, 174). The upregulation of the histone methyltransferase (HMT) SETDB1/SIRT1 is a common feature in HCC. HBx-upregulated miR-29, therefore, acts as a tumor suppressor to modulate the histone methylation transferase SETDB1/SIRT1 leading to a reduction in hepatocarcinogenesis possibly because of a reduction in AKT signaling and/or an increase in pro-apoptotic expression (107, 175). The upregulation of miR-29b has been demonstrated to increase HCC carcinogenesis by repressing SOCS1 expression in the JAK/STAT pathway *via* directly targeting the TET1 DNA demethylation enzyme (176, 177).

#### Innate Immune System

HBx-upregulated miR-29 upregulates LPS induced macrophage activation by repressing AKT1 suppression of a pro-inflammatory response resulting in increased IL-6, IL-1β and NF-_K_B signaling (178, 179). In addition, the upregulation of miR-29 can repress Il-12/Il-23 activation of mature DCs (180) and can influence NK production *via* targeting TBX21/EOMES promotion of IFN-γ (181).

#### Adaptive Immune System

The miR-29a/b cluster plays a crucial role in the thymic production of T-cells, T-cell differentiation and B-cell oncogenic transformation (182, 183) and miR-29 has been cited as a repressor of the immune system because it directly targets IFN-λ in IFN producing immune cells (184). It has also been demonstrated that miR-29 targets IFNAR1 to promote T-cell production (185). In the presence of infection, type 1 IFN signaling and T-BET/EOMES expression modulate Th1:Th2 differentiation. In turn, miR-29a/b directly targets type 1 IFN/T-BET/EOMES thus playing an important role in Th1:Th2 differentiation. Upregulated miR-29a/b blocks type 1 IFN/T-BET/EOMES to promote Th2 expression and reduce Th1 expression. In the HBV-HCC continuum, miR-29a/b is upregulated by the HBx protein suggesting a viral intervention to promote modulate Th1:Th2 differentiation (182). A similar role is played by miR-29a/b when this miRNA is downregulated by intracellular bacteria and fails to modulate type 1 IFN resulting in an imbalance of the production of CD8+ T-cell (182, 183). The upregulation of miR-29 therefore tilts the Th1:Th2 ratio in favor of Th2 expression that also influences B-cell production (184). In addition, the upregulation of miR-29 can repress Il-12/Il-23 activation of Th17 cells (180).

### HBx Epigenetically Dysregulated miR-148/152 Family

#### HBV-HCC Pathogenesis

HBx downregulation of miR-148/152 has a downstream influence on multiple HBV-HCC pathways (**Figure 4**). HBx-downregulated miR-152 influences DNMT1 to silence both the CDH1 and GSTP1 tumor suppressors to promote carcinogenesis. The downregulation of CDH1 increases B-CATENIN in the WNT/B-CATENIN pathway and the downregulation of GSTP1 increases cell proliferation (63). Interestingly HBx activation of DNMT1 also represses cell cycle controls like p16^INK4A^ to promote cell proliferation (186) thus further widening the downstream epigenetic change in HBV-HCC pathways. HBx- downregulated miR-148a fails to suppress HPIP induced upregulation of the mTOR pathway contributing to EMT and increased hepatocarcinogenesis (60). The progression of EMT is also less modulated as a result of this downregulated miRNA failing to repress expression in the MET/SNAIL/EMT pathway contributed to progression of HBV-HCC (61). This downregulated family also fails to regulate the production of B-catenin as a result of the reduced repression of WNT1 signaling (187). HBx-downregulated miR-148a increases SMAD pathway expression by failing to directly regulate TGF-B signaling and by failing to repress USP4 induced TGF-B signaling (188, 189).

### Innate and Adaptive Immune Pathways

The miR-148/152 family play an especially important role modulating the adaptive immune system. In the adaptive immune system this family targets many genes that influence B and T lymphocyte function. This HBx-downregulated miRNA in HBV-HCC targets BCL2L11/TWIST to influence T-cell differentiation and BACH2/MITF to influence B-cell differentiation (190, 191). This family also plays a role in B-cell tolerance and elevated levels of miR-148a which have been noted in autoimmune disorders. In particular, miR-148a targets GADD45, BIM and PTEN that suppress B-cell tolerance (192). This family also targets CAMK11α to suppress MHC class II levels in antigen stimulated DCs that promote T-cell activation. It can thus be hypothesized that if the HBx protein epigenetically downregulates miR-148/152 then this would result in reduced suppression of MHC class II levels in DC activation of T-cells (193). This family, therefore, plays an important role in the innate system as a result of its ability to modulate antigen presenting (APC) DCs which are regarded as the most important class of APC in the innate immune system. DCs, therefore, link the innate and adaptive immune systems (194). This family also modulates IL-6, TNF-α and IFN-β to repress TLR induced DC activation and it can be hypothesized that downregulated miR-148/152 would fail to repress IL-6/TNF-α/IFN-β induced DC expression (194).

### HBx Epigenetically Dysregulated miR-155

#### HBV-HCC Pathogenesis

HBx-upregulated miR-155 is a key epi-miRNA that targets both the HBV-HCC immune and cancer pathways (**Figure 5**). HBx dysregulation of miR-155 can occur due to histone modifications like histone deacetylase inhibitors (HDAC-I) or the repression of polycomb proteins (EZH2) can contribute to upregulated miR-155 expression in HBV-HCC pathways (43, 66, 67). This well researched miRNA is cited as an epigenetic modulator in many cancers including those of the breast, lung and colon (195–197), as well as playing multiple different roles in both the innate and adaptive immune system response (7). In HBV-HCC pathogenesis, upregulated miR-155 typically represses PTEN modulation of AKT/MTOR signaling in the P13K/MAPK pathway that promotes epithelial to mesenchymal transition (EMT) (68, 198). In addition, this miRNA can promote β-catenin expression in the WNT/β-Catenin pathway by repressing the APC/GSK3 destruction complex to thus promoting the transcription of oncogenic proteins like C-MYC (45, 199). This miRNA also represses the SOCS1 tumor suppressor in the JAK/STAT pathway to induce the transcription of CCND1 and c-MYC thus promoting HCC cell proliferation (200, 201). In the TP53 pathway, this key HBx epigenetically upregulated miRNA can repress SOX6 to negate its promotion of p21/Waf1/cip1 modulation of cell cycle controls directly promoting HCC proliferation (6, 69). In a strategy to possibly evade immune system response, this HBx upregulated miR-155 can also subdue HBV replication by blocking the CCAAT/enhancer-binding protein (C/EBP) protein that binds and activates the HBV Enhancer 11/core promoter (199).

#### Innate Immune System

Epigenetically upregulated miR-155 is a key modulator of pro- and anti-inflammatory responses in the innate immune system (202, 203). It is a particularly important miRNA in the modulation of NF-_K_B driven induced myelopoiesis as a result of targeting IRAK1/TRAF6 and SHIP1/SOCS1 respectively (204–206) and also targets CSFR to influence myeloid differentiation (207).

#### Macrophages

SHIP1, an important regulator of the innate system, is a primary target of miR-155 and its repression influences an increase in granulocyte/monocyte cell populations and a reduction in lymphocyte numbers (208, 209) and reduced levels of SHIP1 appears to induce myeloproliferative disorders (208). Interestingly, SHIP-1 is classified as a tumor suppressor in HBV-HCC and reduced levels of SHIP-1 are associated with a poorer prognosis (210). Upregulated mIR-155 in viral infection can induce type 1 IFN induced macrophages *via* by activating the TLR4/MyD88/JNK/NF-_K_B dependent pathway. In order to upregulate TLR4 signaling, upregulated miR-155 can suppress both SHIP1 and SOCSI to block their regulation of downstream TLR signaling directly contributing to increased inflammatory signaling and macrophage activation (208). Furthermore, SOCS1 which regulates type I IFN signaling, is targeted by miR-155 in macrophages (211, 212) and the loss of function of SOCS-1 is a common feature in HCC clearly supporting a hypothesis that HBx-upregulated miR-155 promotes the progression of HBV-HCC (200, 201). Finally, it has been demonstrated that AKT signaling can repress miR-155 in macrophages thus indicating a negative feedback loop to fine-tune TLR signaling (213).

#### Dendritic Cells (DC)

Upregulated-miR-155 modulates the TLR/IL-1 (interleukin-1) inflammation signaling pathway to regulate human monocyte-derived DCs in order to ensure excess damage does not occur (214). TLR/TNF/IFN upregulated miR-155 *via* AP1/BIC plays a significant homeostatic role in monocytepoiesis by repressing PU.1 which activates DC-SIGN, a C-type lectin receptor to increase pathogen cell surface uptake on DCs (207, 215). Decreased DC-SIGN expression in HCC is related to poor prognosis and PU.I has been identified as a metastasis suppressor possibly relating to the impairment of the antigen presenting capabilities of APCs (216).

#### Adaptive Immune System: B-Cells

In the adaptive immune system the epigenetically modulated miR-155 can influence B-cell expression by triggering downstream epigenetic changes. Epigenetically upregulated miR-155 (HDAC-I/EZH2) can repress the expression of an important epigenetic regulator like activation induced cytidine de-aminase (AID) which acts as a HDAC inhibitor that binds to specific immunoglobin genes in the nucleus to induce CSR/SHM/antibody diversification (43). The repression of AID by upregulated miR-155 thus leads to a reduction in CSR/SHM/Plasma B cell diversification thus contributing to reduced ability to synthesize pathogen specific antibodies (217, 218). Interestingly, different studies indicate AID is upregulated in both HBV and HCV induced hepatocarcinogenesis (219, 220). This upregulated miRNA also influences B-cell synthesis by targeting Ship-1 which plays an important role in the regulation of immune cell activation in both the innate and adaptive pathways (221). Epigenetically upregulated miR-155 targets PU-1, a critical transcription factor, to block GC B-cell to Plasma cell transition thereby modulating germinal cell B-cell differentiation into memory cells or plasma cells (222).

#### T-Cells

Epigenetically upregulated miR-155 can target Ship-1 to promote histone building capacity (Phf19) to promote PRC2 expression that promotes histone modifications to repress T-cell senescence and promote CD8+ T-cell expression (67). This miRNA can also modulate IFNγ expression by repressing SHIP1to play a critical role in the reciprocal regulation of CD4+ and CD8+ leukopoiesis (223). MiR-155 also has a role in the generation of exhausted dysfunctional T cells and Fosl2 antagonism of miR-155 can reduce T cell exhaustion during chronic viral infection (224). This upregulated miRNA modulates T helper cell differentiation and the germinal center reaction to synthesize T-cell dependent antibody response. In order to do this, upregulated miR-155 can repress SOCSI to maintain Foxp3^+^ regulatory T-cell (Treg) generation in order to regulate an autoimmune response (225, 226). It can also enhance Treg and Th17 cells differentiation and IL-17A production by targeting SOCS1 (206). A supporting meta study also confirms that the elevated expression of Tregs can be associated with HCC pathogenesis and Treg upregulation is a feature of the HCC tumor microenvironment (227). Conversely, Tregs can also target miR-155 to provide a negative feedback loop to control Treg expression (228). In the Th1/2 differentiation stage upregulated miR-155 can promote Th1 differentiation as a result of targeting C-MAF (229, 230) and an elevated Th17 to Th1 ratio has been associated with tumor progression in HBV-HCC (231). MiR-155 in Th17 cells can also trigger autoimmune inflammation through a signaling network by targeting the Ets1/IL-23/IL-23R pathway (205).

## Clinical Therapeutic Options

The five-year survival rate of advanced HCC remains dismally low and the treatment of advanced stages is limited by a paucity of targeted options despite the fact that HCC cancer pathways and their targeted genes have been well documented. Since the introduction of the multi-kinase inhibitor sorafenib, very little progress has been made in treatment of advanced HCC (232). Novel targeted therapies developed for a range of cancers include the development of immune checkpoint inhibitors like anti-CTLA4 or anti-PD-1/PD-L1 antibodies has introduced new opportunities in clinical oncology (233, 234). Chronic CHB, inflammation and the development of cirrhosis are all hallmarks of HBV-HCC pathogenesis. The question remains as to how miRNA-regulated epigenetic expression can prompt an appropriate immune response.

Our review demonstrates that multiple miRNA can influence epigenetic changes in multiple pathways in HBV-HCC pathogenesis by regulating histone modifications, DNA Methylation and chromatin modelling. For example, we show that HBx- downregulated miR-101 fails to modulate DNMT3A silencing of multiple tumor suppressors in HBV-HCC (24) while simultaneously modulating the expression of macrophages (157), DCs (165), T-cells (166) and B-cells (170). The question remains, however, as to the *in vivo* clinical potential of deploying miR-101 replacement therapy in HBV-HCC. Current trials using HDAC inhibitors to inhibit cell cycle in HCC have been disappointing (235) and to date there has been no attempt to modulate HDAC expression using miRNA in HCC. In a breast cancer study, for instance, HDAC inhibitors reduced tumorigenesis and apoptosis *via* microRNA miR-125a-5p *in vivo* and *in vitro* (236). Epigenetic targeting of EZH2, a histone-lysine-N-methyltransferase and DNMT1 inhibitor reactivated transcriptionally repressed chemokine genes and augmented T cell response in HCC (237). Our review shows that 29 dysregulated miRNA in HBV-HCC (**Table 1**) are both regulated and regulate epigenetic changes offering numerous hypotheses to be tested *in vitro.* In the case of our four HBV-HCC pathways, miR-101 influences PRC2 and DNMT3A silencing, miR-148/152influenced DNMT1/3A silencing, miR-155 repressed PRC2 silencing and miR-29a/b repressed DNMT1/3A silencing in parallel with affecting immune expression in both the innate and adaptive immune systems.

The use of miRNA-led therapeutics is still a work in progress and most likely these therapeutic options would be used as an ancillary form of treatment in support of current options. In theory miRNA-led therapeutics attempt to repress or restore oncogenic and tumor suppressor expression respectively. Currently, miRNA replacement therapy has started investigating whether this could be an adjuvant therapy in support of chemotherapy and radiation (238, 239). This approach relies on the use of synthetic miRNA or miRNA inhibitors to upregulate or downregulate miRNA expression respectively (240). However, this approach has yet to capture the synergistic response generated by multiple miRNA, nor its dynamic homeostatic shifts and this is the puzzle yet to be solved.

## Conclusion

This review attempts to link the epigenetic modifications that influence HBV and host genome expression in HBV-HCC pathogenesis in both the hepatocyte and immune pathways. We examine the interplay between CHB infection, the silencing role of miRNA, epigenetic change, immune system expression and HBV-HCC pathogenesis. In particular, we demonstrate how HBx dysregulated miRNA in HBV-HCC pathogenesis influence and are influenced by epigenetic changes to modulate both the HBV and host genome expression. The paper provides useful insights and potential hypotheses of the complex interplay between host gene targets in the principal cancer and immune pathways as a result of HBx dysregulated miRNA, epigenetic change, HBV-HCC pathogenesis and immune response.

This review paper tries to provide a platform for a wide range of evidence-based hypotheses rather than an (exactly) correct snapshot of the role of miRNA in HBV-HCC pathways. Even though HBV-HCC is a specific sub-type of HCC, there are multiple different classes and stages in which the hypothesized figures would operate to greater or lesser degree. This review should contribute to the point of view that our understanding of miRNA-based pathogenesis is far superior to our current ability to translate this knowledge to improve clinical outcomes.

## Author Contributions

KS conceptualized review article, performed literature review, wrote up first draft including figures and tables. Rewrote drafts 2-4 after fellow author comment. PA assisted re conceptualization of article, reviewed article. CW reviewed article, added text comments. AC reviewed article, added text comments XL reviewed article, added text comments. AK reviewed article, added text comments. All authors contributed to the article and approved the submitted version.

## Funding

JM has received funding within the framework of the Basic Research Program at HSE University.

## Conflict of Interest

The authors declare that the research was conducted in the absence of any commercial or financial relationships that could be construed as a potential conflict of interest.
